# Gamification Strategies in Undergraduate Nursing Education: A Systematic Review Protocol

**DOI:** 10.3390/nursrep15090331

**Published:** 2025-09-09

**Authors:** Raffaele Antonio Elia, Maria Colangelo, Valentina Cerrone, Donato Pace, Vincenzo Andretta

**Affiliations:** 1 Department of Medicine, Surgery and Dentistry, University of Salerno, 84084 Fisciano, Italy; raffaele.elia@ospedalesancarlo.it (R.A.E.); maria.colangelo@ospedalesancarlo.it (M.C.); vandretta@unisa.it (V.A.); 2 Hospital Medical Directorate, Azienda Ospedaliera Regionale San Carlo, 85100 Potenza, Italy; donato.pace@ospedalesancarlo.it

**Keywords:** educational technology, gamification, motivation, nursing education, nursing students, student engagement

## Abstract

**Background/Objectives:** In recent years, the use of gamification has been growing in health education. In undergraduate nursing programs, it aims to enhance motivation, engagement, knowledge retention, and professional competencies. However, the evidence often combining nursing students with other disciplines or focusing on specific tools rather than the broader concept. This systematic review will synthesize the impact of gamification strategies on educational outcomes in undergraduate nursing education. **Methods:** This protocol was written according to PRISMA-P guidelines and is registered in PROSPERO (CRD420251117719). Eligible studies will include randomized controlled trials, quasi-experimental, and observational designs involving undergraduate nursing students exposed to gamification interventions in classroom, online, or clinical training settings. Comparators may include traditional lecture-based instruction or other non-gamified methods. We will search the PubMed, CINAHL, PsycINFO, Web of Science, Scopus, and Google Scholar databases, covering January 2010 to July 2025, without language restrictions. Two reviewers will independently screen studies, extract data, and assess risk of bias using Cochrane RoB-2, ROBINS-I, and JBI Critical Appraisal Tools. Where possible, a meta-analysis will be conducted; otherwise, findings will be synthesized narratively. **Results:** Not applicable; this is a protocol. Findings will be synthesized as specified in the Methods. **Conclusions:** This review will provide a comprehensive synthesis of gamification’s effectiveness in undergraduate nursing education, identifying the most effective strategies and the contexts in which they perform best.

## 1. Introduction

In recent years, there has been a growth of game-based learning in nursing and higher education [1]. Usually summarized as “gamification”, this trend connects to a sizeable body of existing concepts and research in human–computer interaction and game studies, such as serious games, pervasive games, alternate reality games, or playful design [2]. More recent systematic reviews have examined gamification in health professions education, confirming its growing adoption and highlighting heterogeneous effects across contexts [3,4]. Nursing education requires active student engagement as an alternative to traditional knowledge transfer models. Theoretical and procedural content, as well as the development of competencies and skills, must be dynamically integrated, resulting in a complex process that challenges both educators and students [5]. In nursing education, the incorporation of gamification elements can reduce student monotony and passivity, foster active engagement and accelerate the development of professional competencies [6]. The use of gamification has different effects on nursing education, such as control of their learning (autonomy), perceived growth in their skills (competence), and experience collaborative learning (relatedness) [7]. López-Jiménez et al. demonstrated that gamifying an audience response system enhanced both learners’ educational performance and their engagement in anatomy education [8]. Dabbous et al. investigated the use of game-based learning during pharmacy practice experiences, reporting significant improvements in post-test scores and showing that student motivation, measured with the ALMAS scale, predicted learning outcomes [9]. Although game-based learning and gamification are distinct approaches, this study illustrates the potential of playful educational strategies in health professions, reinforcing the need for a review specifically focused on gamification in undergraduate nursing education. In another study involving nursing students, participants were first assessed for their baseline ability to interpret electrocardiograms (ECGs). Each group then received four structured training sessions, and variables of interest were re-evaluated two weeks after the intervention, revealing improved diagnostic accuracy and retention of skills [10]. However, Sanz-Martos et al. [11] evaluated a gamification session within nursing education and found that both student satisfaction and knowledge scores improved significantly (*p* < 0.001) following gamified lab-based activities.

In recent years, several systematic reviews have investigated the use of gamification in education, but many have included heterogeneous populations, including students from non-healthcare disciplines or education levels other than undergraduate [3,4,12,13]. This approach has produced results that are difficult to generalize to nursing education, where the training needs and required skills are specific and highly professionalizing. Furthermore, the available reviews have not always clearly distinguished between the different types of gamified strategies, nor have they analyzed motivational and affective outcomes in addition to cognitive ones. A preliminary search conducted in JBI, OSF, and PROSPERO databases did not identify any existing systematic reviews on this topic, which supports the rationale for the present protocol. This systematic review aims to fill these gaps by providing a targeted synthesis of the available evidence and a critical analysis of the barriers and facilitators to implementing such interventions in nursing degree programs.

The objective of this systematic review is to assess the impact of gamification strategies on educational outcomes in undergraduate nursing education. Specifically, the review will examine how gamification affects student learning, engagement, motivation, and knowledge retention, and will identify any reported challenges or barriers related to its implementation in academic settings.

## 2. Materials and Methods

### 2.1. Protocol and Registration

This systematic review protocol will be developed in accordance with the Preferred Reporting Items for Systematic Review and Meta-Analysis Protocols (PRISMA-P) guidelines [14]. The protocol has been registered in the International Prospective Register of Systematic Reviews (PROSPERO; ID: CRD420251117719, available online at: https://www.crd.york.ac.uk/PROSPERO/view/CRD420251117719) on 10 August 2025 [15].

Any protocol amendments will be updated in PROSPERO with a date-stamped record prior to data extraction and analysis.

### 2.2. Eligibility Criteria

#### 2.2.1. Population

Studies will be eligible if they involve undergraduate nursing students enrolled in formal nursing education programs (e.g., bachelor’s or three-year degree courses) participating in educational interventions that incorporate gamification strategies, regardless of the setting (classroom-based, online, or clinical training). Studies involving non-nursing students (e.g., medical, pharmacy, or allied health) or conducted in unrelated educational contexts (e.g., secondary education, non-healthcare disciplines) will be excluded.

#### 2.2.2. Intervention(s) or Exposure(s)

Eligible interventions will be educational strategies explicitly integrating gamification elements—such as points, badges, leaderboards, narratives, and challenges—into undergraduate nursing programs. We define gamification as the integration of game design elements (e.g., points, badges, leaderboards, narrative/quests, levels, feedback loops, cooperative/competitive mechanics) into non-game educational activities. Serious games will be included only if nursing-specific data are reported and will be analyzed separately. Stand-alone serious games without gamification elements will be excluded. Gamification may be implemented in classroom teaching, online modules, or clinical simulations. Studies will be excluded if they do not incorporate gamification elements or if the approach is unrelated to nursing education.

#### 2.2.3. Comparator(s) or Control(s)

When present, comparators may include traditional, non-gamified educational approaches (e.g., lecture-based instruction, standard classroom teaching). Studies comparing two gamified interventions will be excluded.

#### 2.2.4. Study Design

Eligible study designs include randomized controlled trials (RCTs), quasi-experimental studies, nonrandomized controlled studies, and observational designs (cross-sectional and cohort studies). Editorials, opinion papers, reviews, protocols, and conference abstracts without full text will be excluded.

#### 2.2.5. Context

Eligible studies must be conducted in academic or formal educational settings involving undergraduate nursing students (e.g., universities, nursing schools).

### 2.3. Information Sources and Search Strategy

The main databases to be searched will include CINAHL (via EBSCOhost), PsycINFO (via EBSCOhost), PubMed (MEDLINE), Web of Science Core Collection, and Scopus. Google Scholar will also be consulted, and only the first 200 results sorted by relevance will be screened to ensure feasibility. Search strategies have been harmonized across databases to ensure consistency; Boolean operators (AND, OR) were used systematically, while restrictive operators such as NOT were avoided unless clearly justified.

No language restrictions will be applied. Non-English studies will be translated using professional translation services or validated translation software, with verification by bilingual reviewers to ensure accuracy. The search will cover the period from 1 January 2010 to 31 July 2025. The time frame is to 15 years to capture the emergence and development of gamification in nursing education, because the concept gained relevance in higher education literature after 2010. Search strategies will be adapted for each database. Combinations of keywords and MeSH terms related to “nursing students”, “gamification”, “educational games”, “motivation”, “engagement”, and “knowledge retention” will be used. Search strings were harmonized across databases to reduce redundancy while ensuring sensitivity. Related terms (e.g., ‘nursing student,’ ‘undergraduate nursing,’ ‘bachelor nursing’) were included to account for variations in indexing.

**Figure 1:**
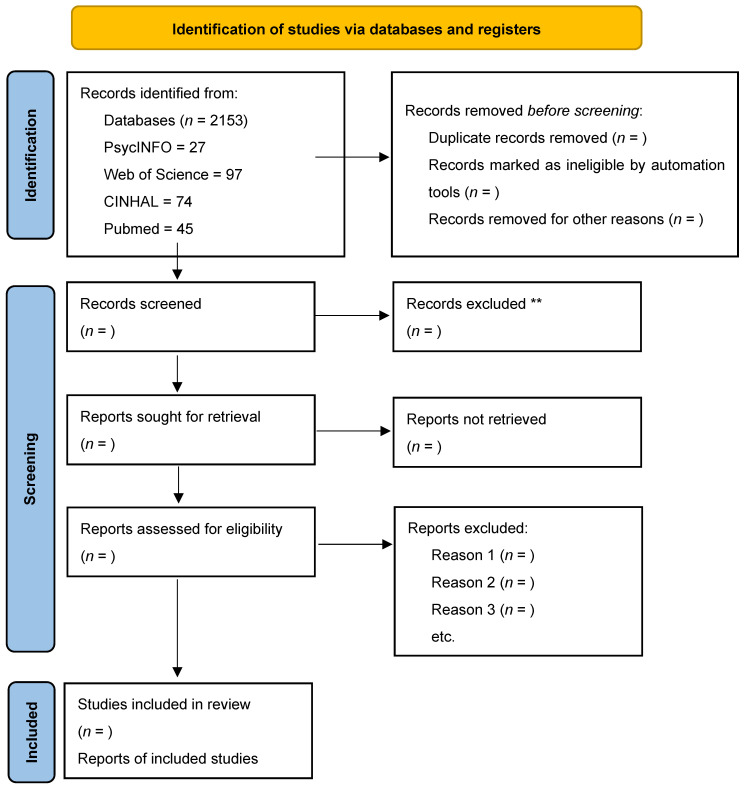
PRISMA 2020 flow diagram for new systematic reviews which included searches of databases (n values will be added once the screening and selection process has been completed). ** Indicates that further details (such as exclusion reasons) will be added during the full review process.

The study selection process will be documented using the PRISMA 2020 flow diagram (Figure 1). At present, the diagram reports the number of records retrieved in preliminary searches (Table 1). The diagram will be completed with counts of screened, excluded, and included studies during the full review process.

The search strategy was developed and refined with the support of an experienced medical librarian to optimize both sensitivity and specificity [16].

### 2.4. Study Selection

Search results will be imported into Zotero for reference management and duplicate removal [17]. Two reviewers (R.A.E. and M.C.) will independently screen titles and abstracts, followed by full-text assessments of potentially eligible studies. Disagreements will be resolved by consensus or by consulting a third reviewer (V.C.).

Inter-rater reliability between the two independent reviewers will be assessed using Cohen’s Kappa statistic, with disagreements resolved through discussion or adjudication by a third reviewer.

### 2.5. Data Extraction

A standardized data extraction form, developed a priori, will be used to collect information on study characteristics (e.g., authors, year, country, setting, design), participant demographics, intervention characteristics (type of gamification, game elements, duration, frequency), comparators, outcomes assessed, measurement instruments, and key findings. The form will be piloted on a sample of studies and refined as necessary. Data will be extracted independently by at least two reviewers (or a person/machine combination) with a process to resolve any discrepancies.

### 2.6. Risk of Bias Assessment

Risk of bias will be assessed using the following:

- Cochrane RoB-2 for RCTs [18];
- ROBINS-I for nonrandomized studies [19];
- JBI Critical Appraisal Tools for cross-sectional and descriptive studies [20];
- GRADE approach for certainty of evidence for each outcome [21];
- GRADE-CERQual framework for qualitative evidence [22].

Two reviewers will perform assessments independently.

### 2.7. Outcomes

Primary outcome:

The primary outcome will be academic performance/knowledge retention. Retention will be categorized according to time windows: immediate (≤1 week), short-term (1–4 weeks), medium-term (1–3 months), and long-term (>3 months). Academic performance will be assessed through validated or standardized tests and examinations, with effect sizes extracted as mean difference or standardized mean difference.

Secondary outcomes:

- Motivation and satisfaction, measured through validated questionnaires such as the *Gameful Experience Scale* (*GAMEX*) or Likert-style items.
- Engagement and critical thinking, assessed through GAMEX subscales (e.g., Absorption, Creative Thinking) or validated instruments such as the *SEGiNAS* scale.
- Implementation challenges, defined as barriers and facilitators reported during the design, delivery, or evaluation of gamification strategies. These qualitative findings will be analyzed through thematic synthesis, and their confidence will be appraised using the GRADE-CERQual approach.

### 2.8. Data Synthesis

Where studies are sufficiently homogeneous in terms of design, population, and outcome measures, a meta-analysis will be conducted, using the Review Manager (RevMan, Version 5.4.1, Cochrane Collaboration, Copenhagen, Denmark). The primary meta-analyses will include randomized controlled trials (RCTs) and controlled quasi-experimental studies. For continuous outcomes, mean differences or standardized mean differences with 95% confidence intervals will be calculated using a random-effects model to account for expected heterogeneity. Observational designs (cross-sectional, cohort, descriptive studies) will be narratively synthesized, and, if feasible, analyzed separately. Studies that directly compare two gamified interventions (e.g., gamified quiz vs. gamified simulation) will be synthesized narratively as a distinct comparison set. When meta-analysis is not feasible, a structured narrative synthesis will be provided. Qualitative findings regarding implementation challenges will be analyzed using a thematic synthesis approach. The methodological quality of qualitative studies will be appraised using the JBI Critical Appraisal Tools (2020 version), and the confidence in the evidence will be assessed using the GRADE-CERQual (2018 guidance) approach.

### 2.9. Certainty of Evidence

The certainty of evidence will be evaluated using the GRADE approach for each primary outcome, considering risk of bias, inconsistency, indirectness, imprecision, and publication bias.

### 2.10. Ethics and Dissemination

As this review is based solely on published studies, ethical approval is not required. The results will be disseminated via a peer-reviewed publication and conference presentations.

## 3. Results

It is anticipated that the systematic review will identify a range of gamification strategies implemented in undergraduate nursing education, such as gamified flipped classrooms, laboratory-based simulations, interactive digital quizzes, and serious games. Based on the existing literature, these interventions are likely to be associated with improvements in learning motivation, preparedness, theoretical knowledge, and self-confidence [23]. However, it is also anticipated that some studies will report limited or no effects on practical skill performance, suggesting that the benefits of gamification may be more pronounced for cognitive and motivational outcomes than for psychomotor skills.

The review will likely find that gamified laboratory sessions can increase both student satisfaction and knowledge scores, while also fostering critical thinking and facilitating the transfer of theory into practice. Qualitative evidence is expected to highlight additional benefits in affective learning domains, such as the development of empathy, reflective capacity, and professional identity [9]. For example, gamified activities in mental health nursing may be reported to enhance students’ competencies, confidence, and engagement with complex patient care scenarios [24].

Moreover, this review will assess whether evidence from related health education fields can be synthesized to estimate the impact of gamification on academic achievement and knowledge retention [11]. The review will also explore how the magnitude of effects may differ according to the type of gamification design, the learning context, and the assessment methods used.

## 4. Discussion

The current literature on gamification in nursing education is characterized by heterogeneity, with differences in populations, designs, outcomes, and measurement instruments, thus making it difficult to draw definitive conclusions about the effectiveness of gamification, specifically in undergraduate nursing education.

This systematic review aims to fill this gap by providing a targeted and structured synthesis of the available evidence, including cognitive, motivational, and affective outcomes, as well as the challenges associated with the implementation of gamification strategies. Differences in the effectiveness of interventions will be explored according to type (e.g., quizzes with leaderboards, simulation-based gamification, virtual reality, and board games). If sufficient homogeneous data are available, pooled effect sizes will be calculated using a random-effects model to estimate the magnitude of gamification effects. For outcomes not amenable to statistical pooling, a structured narrative synthesis will be conducted, integrating both quantitative and qualitative findings.

## 5. Conclusions

This review will synthesize the available evidence on the efficacy of gamification in nursing education, highlighting its potential to improve motivation, involvement, knowledge retention, and the development of professional competencies. The expected results will help to identify the most effective strategies and the contexts in which they work better, offering educators useful tools to design innovative, student-centered educational approaches, and to orient future research in this field.

## Figures and Tables

**Table 1:** Presents the search strings and the number of results retrieved from each database. The asterisk (“*”) is used as a truncation symbol to retrieve all possible word variants (e.g., “student *” = “student” OR “students”).

Database	Interface	Search String	Results
**PsycINFO**	EBSCOhost	((“nursing student” OR “nursing students”) OR “undergraduate nursing” OR “bachelor nursing”) AND (“game-based learning” OR gamification OR “educational games” OR “serious games”) AND (education OR “nursing education” OR “academic performance” OR “knowledge retention” OR motivation OR engagement)	27
**Web of Science**	Web of Science Core Collection	TS = (“nursing student *” OR “undergraduate nursing”) AND TS = (gamification OR “game-based learning” OR “educational game *” OR “serious game *”) AND TS = (“motivation” OR “engagement” OR “knowledge retention” OR “academic performance”)	97
**CINAHL**	EBSCOhost	(“nursing student *” OR “undergraduate nursing” OR “bachelor nursing”) AND (“gamification” OR “game-based learning” OR “educational games” OR “serious games”) AND (“motivation” OR “engagement” OR “knowledge retention” OR “academic performance”) NOT (“medical students” OR “pharmacy students” OR “allied health”)	74
**PubMed**	MEDLINE	(“nursing students” [tiab] OR “nursing student” [tiab] OR “undergraduate nursing” [tiab]) AND (“gamification” [tiab] OR “game-based learning” [tiab] OR “educational games” [tiab] OR “serious games” [tiab]) AND (“motivation” [MeSH Terms] OR “motivation” [tiab] OR “engagement” [tiab] OR “academic performance” [tiab] OR “knowledge retention” [tiab])	45
**Scopus**	Elsevier	TITLE-ABS-KEY(“nursing students” OR “undergraduate nursing” OR “bachelor nursing”) AND TITLE-ABS-KEY(“gamification” OR “game-based learning” OR “educational games” OR “serious games”) AND TITLE-ABS-KEY(“motivation” OR “engagement” OR “knowledge retention” OR “academic performance”)	120
**Google Scholar**	scholar.google.com	“undergraduate nursing students” AND (“gamification” OR “game-based learning”) AND (“motivation” OR “engagement”)	1790

## Data Availability

No new data were created or analyzed in this study. Data sharing is not applicable to this article.

## Public Involvement Statement

There was no public involvement in any aspect of this research.

## Guidelines and Standards Statement

This manuscript was drafted against the Preferred Reporting Items for Systematic Review and Meta-Analysis Protocols (PRISMA-P) 2015 statement for systematic review protocols.

## Abbreviations

The following abbreviations are used in this manuscript:

Abbreviation	Full term
PRISMA-P	Preferred Reporting Items for Systematic Review and Meta-Analysis Protocols
CINAHL	Cumulative Index to Nursing and Allied Health Literature
WOS	Web of Science
GAMEX	Gameful Experience Scale
JBI	Joanna Briggs Institute
RCT	Randomized Controlled Trial
ROBINS-I	Risk Of Bias In Non-randomized Studies—of Interventions
GRADE	Grading of Recommendations, Assessment, Development, and Evaluations
